# Sleep deprivation alters utilization of negative feedback in risky decision-making

**DOI:** 10.3389/fpsyt.2024.1307408

**Published:** 2024-11-19

**Authors:** Wenhao Xu, Lubin Wang, Liu Yang, Yuyang Zhu, Pinhong Chen

**Affiliations:** ^1^ Beijing Institute of Basic Medical Sciences, Beijing, China; ^2^ Air Force Medical Center, Air Force Medical University, Beijing, China

**Keywords:** sleep deprivation, risky decision making, vigilant attention, emotion, negative feedback

## Abstract

**Background:**

Sleep loss has sometimes catastrophic effects on risky decision-making. However, it is unknown to what extent such deficits are exacerbated with increasing duration of sleep deprivation (SD) and whether sustained vigilant attention mediates this sleep deprivation-induced deficit.

**Methods:**

The present study aimed to investigate the effect of 36 hours of SD on 37 male college students’ arousal, emotion, vigilant attention, and risky decision-making, using the Psychomotor Vigilance Test, the Game of Dice Task, and scales assessing fatigue, sleep, and emotions.

**Results:**

Compared to baseline, SD significantly increased sleepiness, fatigue, and negative emotions, decreased positive emotions and vigilant attention, and led to a shift toward risky decision-making, and these effects often appeared 15 or 20 hours after SD. Interestingly, participants’ ability to employ positive feedback was maintained, whereas their performance to utilize negative feedback was impaired even after 8 hours of sleep deprivation. Meanwhile, vigilant attention acted as a mediator between SD and risky decision-making (z = -1.97, 95% [-6.00, -0.30]).

**Discussion:**

These results suggest that sleep-deprived individuals are unable to use negative feedback to optimize their judgments, which may account for their poor decision-making under risk.

## 1 Introduction

Sleep, as the basic physiology of the body for a healthy life, can not only promote the body’s growth and development but also maintain the body’s regular work and life (1–3). However, for a variety of reasons in real life, our sleep quality deteriorates and our sleep time decreases (4, 5), arousing widespread concern in society and becoming a global problem (6). Sleep deprivation (SD) and sleep loss have been shown in many studies to result in a variety of psychological and cognitive capabilities changes (2, 7–9). Meanwhile, acute SD has been shown to significantly impair various cognitive capabilities, including working memory, cognitive control, and emotional regulation (10–13). Research indicates that just one night without sleep could age the brain by 1 to 2 years (14). Therefore, increasing SD causes changes in individual physiology, psychology, and behavior, which is worth exploring.

We focused on how SD influenced risky decision-making, which is a decision under uncertain conditions. The study of the Balloon Analog Risk Task (BART) found that participants with moderate sleepiness took longer to complete the BART, pumped more balloons, and exploded more balloons (15). When compared to controls, people with SD are less able to learn to choose from the advantageous decks on the Iowa Gambling Task (IGT), resulting in inferior decision-making ability (16). Previous research has demonstrated that risk perception (17, 18) and risk tolerance (19) influence individual decision-making. Risk perception is an individual’s perception and comprehension of risk, including uncertainty estimation and confidence in the calculation (20). Individuals are more sensitive to benefits and pay more attention to maximizing gains after SD, whereas their sensitivity to losses is reduced; that is, SD diminishes an individual’s ability to recognize risk (21, 22). Risk tolerance is defined as an individual’s willingness to take risks in order to attain a given goal (23). Although sleep-deprived individuals perceive high risk, high yield will increase the attractiveness of options and raise the threshold of individual risk tolerance, leading to individuals being more inclined to seek out risk in risky decision-making. Currently, the tools we commonly used to measure risky decisions include BART, IGT, and Game of Dice Task (GDT). The GDT is a gambling task that has defined rules for gains and losses as well as fixed winning odds (24), which is used in the study of SD on risky decision-making (25).

Studies have shown that SD decreases attention span and reduces vigilant attention (2, 26, 27). Vigilant attention refers to the ability of an individual to maintain a stable level of alertness over a period of time and is the foundation of many higher cognitive capabilities (28, 29). After 24 hours of SD, subjects’ attention is distracted, so they were unable to complete the alert mission (30). The Psychomotor Vigilance Test (PVT) is highly sensitive to behavioral alertness deficits due to sleep loss (31) in both acute SD and partial SD (32). Drummond et al. (33) found that subjects’ performance in the PVT decreased significantly after 36 hours of SD. The PVT score was negatively correlated with the functional connection strength of the default network and positively correlated with the functional connection strength of the prefrontal cortex, parietal lobe, and subcortical structures (34, 35). Studies of patients with attention-deficit/hyperactivity disorder (ADHD) have confirmed that vigilant attention has been found to heavily influence inappropriate or risky decisions (36–38). Training attention improves decision-making in individuals with elevated self-reported depressive symptoms (39).

SD is also related to an individual’s mental states and emotions. Whether it is subjective scale (40), heart rate variability analysis (41, 42), or electroencephalograph (EEG) analysis (43), SD increased fatigue and sleepiness. Both sleep quality and sleepiness are related to fatigue (44). An earlier SD study by Kaida and Niki (45), showed an increase in negative affect (POMS: the POMS subscale scores of sleepiness, confusion, fatigue, and anger) and a decrease in positive affect (the POMS subscale scores of vigor). Functional magnetic resonance imaging (fMRI) studies have shown that sleep-deprived people have altered emotional brain networks, mainly in the limbic system (46), specifically thalamic (47, 48).

According to a summary of previous studies, few studies collected data multiple times due to the complexity of the SD process and the negative emotions of the participants. In this study, similar to constant routine (CR) design (49, 50), participants were subjected to 36 hours of SD, and data collection was conducted seven times. To investigate the relationship between SD and risky decision-making and the role of vigilant attention, we conducted an in-laboratory SD study. Our hypotheses are: (1) as SD increased, sleepiness, fatigue, and subjective negative mood increased significantly, and positive emotions decreased; (2) as SD increased, PVT response times (RTs) increased and GDT scores decreased, so vigilant attention and risky decision-making deteriorated; (3) SD affects risky decision-making by reducing vigilant attention.

## 2 Methods

### 2.1 Participants

A total of 37 college male students were recruited through online advertisements in Beijing, China. Considering the sleep pattern changes over the menstrual cycle phase in women, only male subjects were included in our study. A simple interview survey was conducted by an experienced clinician, and participants having a history of mental health disorders, neurological disorders, or organic diseases were not allowed to participate. None of the participants mentioned having experienced identical diseases in the past. Before coming to the laboratory, participants were instructed to keep a regular sleep schedule and refrain from alcohol, caffeine, and chocolate intake for at least 1 week before the study in order to establish a typical sleep pattern. We measured age (23.18 ± 1.98) and BMI (21.50 ± 3.04), reflecting relatively homogenous. Meanwhile, we also explored potential correlations between age, BMI, levels of sleepiness, fatigue, and vigilant attention. Our findings revealed no significant correlations among these variables. Approved by the Research Ethics Committee of the Beijing Institute of Basic Medical Sciences, the protocol of our study was explained to all the participants, and written informed consent was administered prior to the experiment.

### 2.2 Subjective scale

#### 2.2.1 The Stanford sleepiness scale

The Stanford Sleepiness Scale (SSS) was designed to quantify subjective sleepiness levels in studies of sleep disorders and SD (51). It consists of a seven-point scale of identical intervals from 1 (“feeling active and vital; alert; wide awake”) to 7 (“almost in reverie; sleep onset soon; lost in struggle to remain awake”). A higher score on the SSS is associated with poorer sleep quality (52). The scale is simple and easy to evaluate and has been widely used in SD studies (53, 54). SSS measures energetic arousal (ranging from feeling sleepy to feeling awake) (55). To measure instant-moment sleepiness multiple times (56), we chose SSS. The Cronbach’s alphas in our study were 0.83.

#### 2.2.2 Measurement of subjective pressure and fatigue

Similar to visual analogue scale (VAS), The questionnaire has a total of two questions and aims to briefly assess stress and fatigue. How stressed and fatigued participants felt were rated on a 9-point scale ranging from 1 (completely relaxed/awake), 5 (between relaxed and nervous/between awake and fatigue), and 9 (absolutely nervous/fatigue).

#### 2.2.3 The abbreviated profile of mood states

The abbreviated profile of mood states is an adjective checklist aimed at measuring the transient emotional states of athletes as well as other groups (57). The instrument contained 40 adjectives referring to seven mood states: five negative emotional states (fatigue, anger, confusion, tension, and depression) and two positive emotional states (vigor and esteem-related affect), whose reliability ranges from 0.60 to 0.82 in China. Participants rated their current feelings on a 5-point scale from 0 (not at all), 1 (a little), 2 (moderately), 3 (quite a bit), and 4 (extremely). The higher the POMS, the worse the mental health. This questionnaire can be effectively used to study the emotional state of normal people. The POMS could include tense arousal (ranging from feeling calm to feeling nervous). The Cronbach’s alphas in the study were 0.79.

### 2.3 PVT

One of the most commonly utilized measures in sleep research is PVT (58), a measure of vigilant attention that requires participants to rapidly respond to visual cues randomly presented within specified interstimulus intervals (ISIs) without incorrectly responding when no stimulus is present. The PVT duration was 5 minutes in the study, including 50 trials. The experimental process was as follows: a visual stimulus was presented at the center of the screen, with a time interval ranging from 2 to 10 s. Participants were instructed to remain attentive and were required to press the left mouse button as quickly as possible upon the appearance of the gray square stimulus on the computer screen, without feedback. When the participant did not make a key response within 1s of the appearance of the stimulus, the stimulus would automatically disappear and move to the next trial. Reaction times exceeding 500 ms are classified as missed trials, whereas those below 100 ms are designated as false starts. The level of sustained attention for each subject was evaluated by calculating the mean of the remaining trials’ reaction times, excluding the aforementioned missed trials and false starts.

### 2.4 GDT

The GDT can be considered a well-established task to measure decision-making in laboratory situations, showing good criterion validity and good discriminant validity (25, 59). The computerized GDT was designed to assess decision-making under explicit and stable rules for gains and losses, as well as winning probabilities (60). Participants start with a balance of 1000 yuan and are instructed to win as much money and lose as little money as they can. In a total of 18 rounds, one die is thrown, and participants each time are supposed to guess the correct number by choosing a single number or a combination of numbers (two, three, or four numbers), which is associated with different probabilities for gains and losses (winning probabilities 1:6, 2:6, 3:6, and 4:6, and gain/losses 1000 RMB, 500 RMB, 200 RMB, and 100 RMB, respectively). If the result of each throw matches the chosen dice number or any combination of numbers, participants win the specified amount; otherwise, they lose the same amount of money. As winning probabilities are below 50%, one single number and combinations of two numbers are considered disadvantageous decision-making alternatives, while combinations of three or four numbers are regarded as advantageous alternatives as the winning probabilities are at 50% or above. After participants makes their choice, the corresponding financial gain or loss is displayed for 500ms in the corner. In our study, risk-taking propensity was estimated by the GDT net score, the frequency of positive feedback, and the frequency of negative feedback. The GDT net score is typically calculated by the number of advantageous choices minus the number of disadvantageous choices, with a positive net score indicating superior performance. Furthermore, the frequency of positive feedback refers to the number of people persisting in an advantageous alternative after a win following an advantageous choice, and the frequency of negative feedback indicates the number of people shifting to an advantageous option after a loss following a disadvantageous choice, as in previous studies.

### 2.5 Study procedure

All participants were asked to maintain sleep diaries one week prior to and throughout the study to ensure that the volunteers went to bed no later than 12:00 AM (midnight) and work no later than 9:00 AM. Every two participants were invited to our lab at a time. Before the formal experiment began, the participants had to understand and master the decision-making task by practice and reported task rules, so the effect of practice was eliminated. They keep them awake 36 hours a day from 7:00 AM. Sleepiness, subjective pressure, fatigue, affect response, PVT response, and decision-making were checked seven times, which are the baseline, 8 hours, 15 hours, 20 hours, 25 hours, 32 hours, 36 hours during SD. For the other time, participants were allowed to engage in some non-strenuous activities, such as reading and talking with someone in the laboratory illuminated with standard office lighting (~500 lx). Three meals a day were provided for them by our nutritionists in order to reduce the impact of food intake on the circadian clock.

### 2.6 Statistical analysis

All data were analyzed using IBM SPSS statistical software (version 22.0) and Mplus 8.3 for Windows (SPSS, Chicago, IL, United States), with two-tailed P values<.05 considered significant.

#### 2.6.1 Correlation analysis

Analyses of correlation and partial correlation were performed to confirm the relationship between each variable.

#### 2.6.2 Mixed linear model

In order to reduce missing data due to repeated measurements, we used a mixed linear model for analysis. AR (1) (first-order autoregressive covariance structure) was selected as the optimal structure to evaluate the difference between different indicators at different times. Test time was a fixed effect, and different mental states and subjective moods, PVT-RTs, and GDT scores were dependent variables with a random intercept. The least significant difference (LSD) was used to make multiple postmortem comparisons.

#### 2.6.3 Mediation analysis

Potential mediators of the change in SD were examined in SPSS using Model 1 in the MEMORE macro (Version 2.1; 61), which is specifically designed to assess mediation in two-instance repeated measures research designs. This procedure computes a pre-post-difference score and then determines whether the mediator of interest predicts that difference (62).

## 3 Results

### 3.1 Fatigue, sleepiness, and pressure

**Figure 1:**
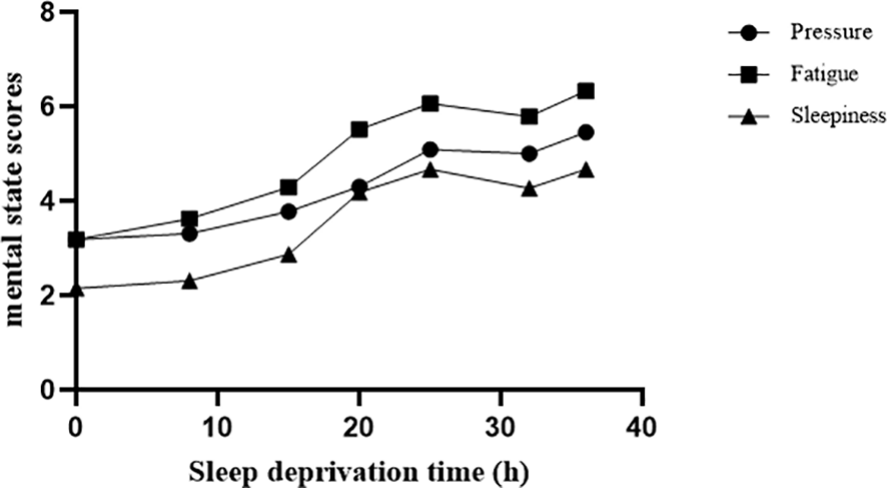
Different mental states changed with SD. We measured 7 time points: baseline, 8 hours, 15 hours, 20 hours, 25 hours, 32 hours, and 36 hours during SD. Pressure, fatigue, and sleepiness increased significantly with increasing duration of SD.

The difference in weariness was found to be statistically significant at seven time periods [*F*(6,111)=24.49, *p*<0.001]. Compared to baseline, the 15th, 20th, 25th, 32nd, and 36th hours were statistically significant (*ps<*0.01), with the 36th hour having the highest significance (t=9.56, 0.001). The findings revealed that the variations in sleepiness at various time points were statistically significant [F(6,137)=15.60, *p*<0.001]. Compared to baseline, the 15th, 20th, 25th, 32nd, and 36th hours were statistically significant (*ps*<0.01), with the 36th hour having the highest significance (t=8.15, *p*<0.001). The pressure difference between time points was statistically significant [F(6,160)=6.13, *p*<0.001]. The 15th, 20th, 25th, 32nd, and 36th hours were statistically significant (*ps*<0.01) when compared to the baseline. As shown in **Figure 1**, fatigue, sleepiness and pressure increased with the duration of SD. Meanwhile, the Pearson correlation coefficients of sleepiness and fatigue were significant at different time points (*ps*<0.01).

### 3.2 Affect response

**Figure 2:**
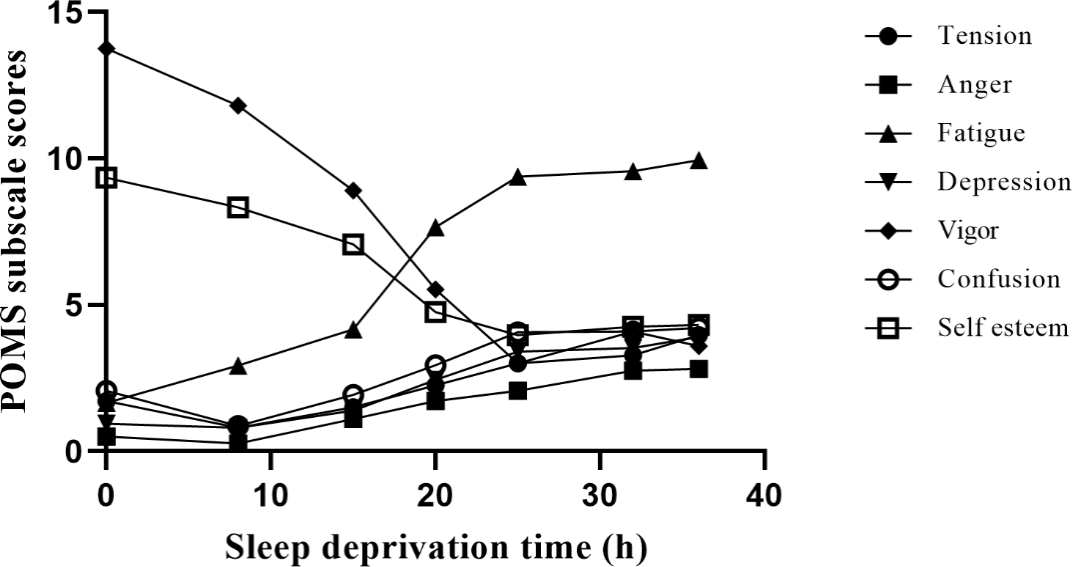
Different moods change with SD. Negative emotion includes tension, fatigue, anger, depression, and confusion; positive affection includes vigor and esteem-related affect. We measured 7 time points, including baseline, 8 hours, 15 hours, 20 hours, 25 hours, 32 hours, and 36 hours during SD. Among them, vigor and self-esteem showed a downward trend, while other moods showed an upward trend.

As shown in **Figure 2**, the mixed liner model of POMS revealed an increase of five subscales related to negative emotion (tension, fatigue, anger, depression, and confusion; *ps*<0.01) and a decrease of two subscales of positive affection (vigor and esteem-related affect; *ps*<0.01) with the duration of SD. In negative emotions, compared to baseline, fatigue was significant from the 8th hour, anger from the 15th hour, depression from the 20th hour, and confusion and tension from the 25th hour (*ps*<0.05). Meanwhile, compared to baseline, vigor was significant from the 8th hour, and self-esteem was significant from the 15th hour (*ps*<0.05). Pearson correlation coefficients of five subscales related to negative emotion (e.g., anger and fatigue) were significant after 36 hours of SD (*ps*<0.01), and the Pearson correlation coefficient of two subscales related to positive emotion (i.e., vigor and tension) was significant after 36 hours of SD (*p*<0.01).

### 3.3 PVT

**Figure 3:**
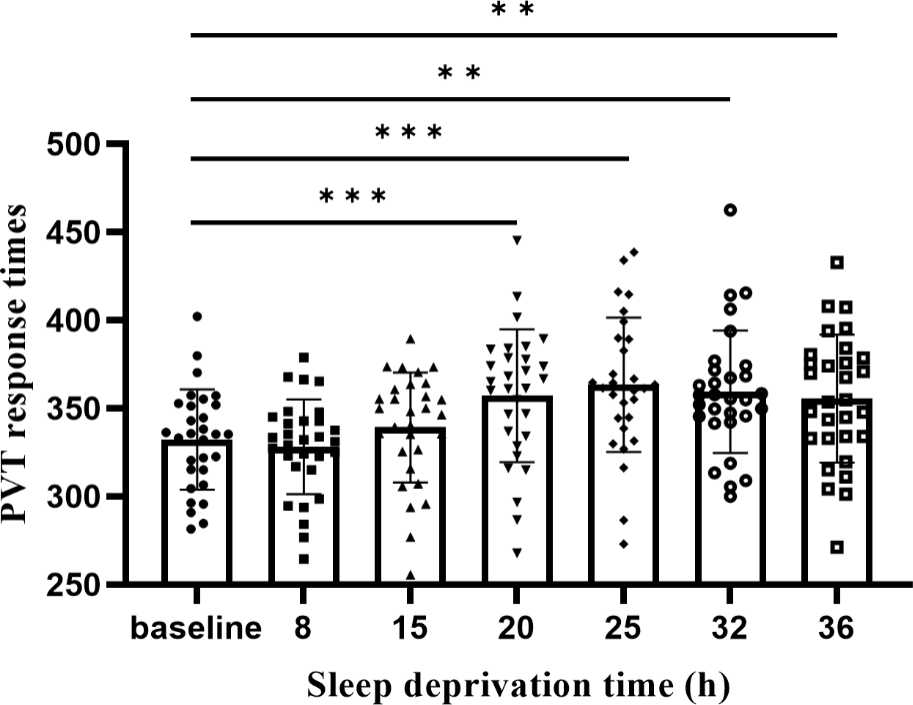
With SD, the response to the PVT changes. From 20 hours of SD, there were significant differences in the duration of deprivation from baseline. **p<01, ***p<.001.

As shown in **Figure 3**, the result showed that the differences between the mean PVT RTs at different time points were statistically significant [F(6,181) = 5.20, *p*<0.001]. The differences between the 20th, 25th, 32nd, and 36th hours were statistically significant compared with the baseline (*ps*<0.01). There was no difference between the 20th, 25th, 32nd, and 36th hours (*ps*> 0.05).

### 3.4 GDT

**Figure 4:**
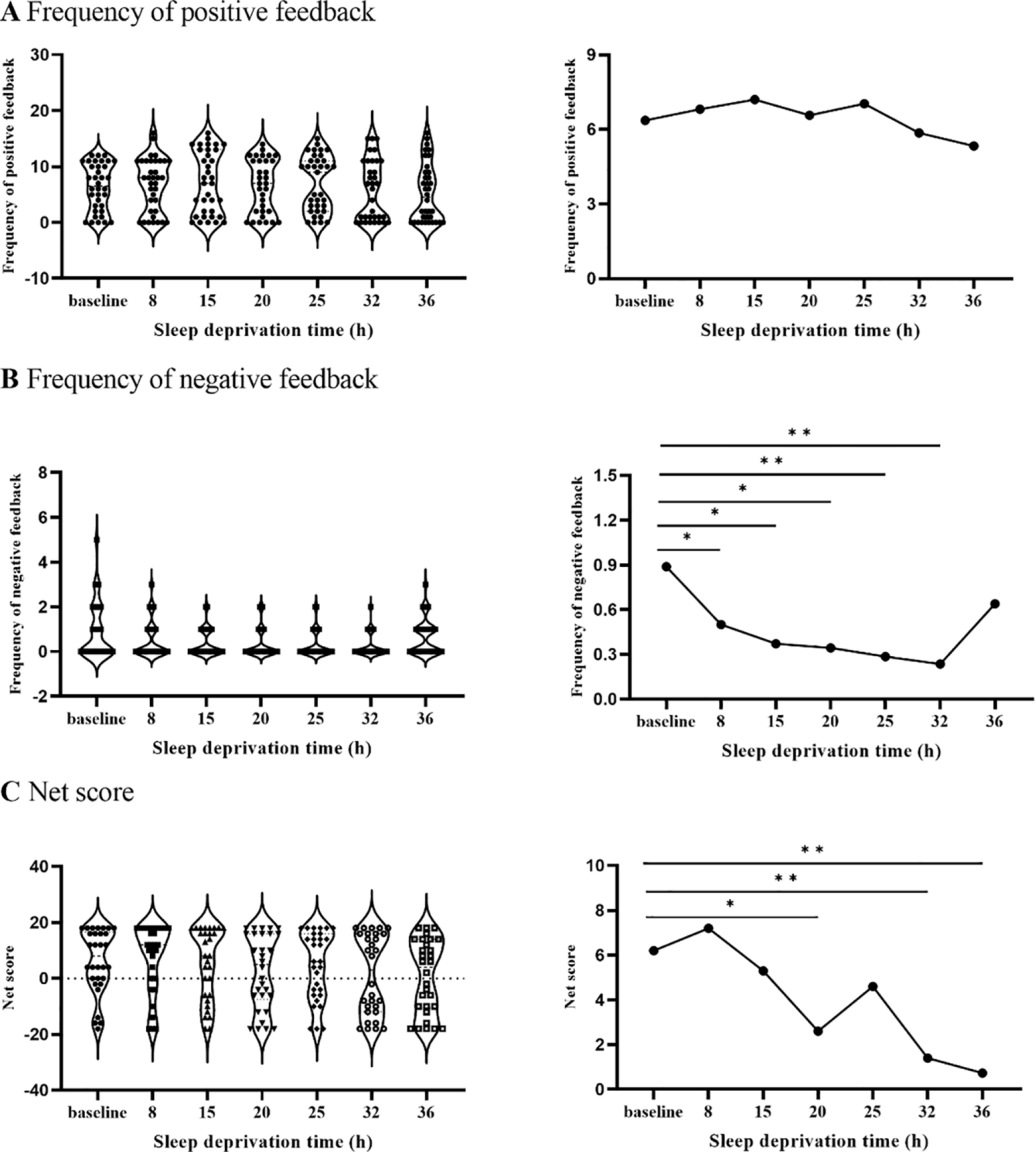
The changing trend of frequency of positive and negative feedback and net score with SD. **(A)** illustrates that the frequency of positive feedback did not change after SD. **(B)** represents that the frequency of positive feedback decreased after 8 hours of SD. **(C)** illustrates that the net score decreased significantly after 20 hours of SD. *p<.05, **p<01.

The results showed that there were statistically significant differences in the frequency of negative feedback from GDT at different time points [F(6,137)=3.01, *p*<0.01]. When compared to the baseline, the differences between the 8th, 15th, 20th, 25th, and 32nd hours were statistically significant (*ps*<0.05, **Figure 4**). There was no difference (*ps*> 0.05) between the 8th, 15th, 20th, 25th, and 32nd hours.

The result showed that the differences between the net scores of GDT at different time points were statistically significant [F(6,130) = 3.15, p<0.01]. The differences between the 20th, 32nd, and 36th hours were statistically significant compared with the baseline (*ps*<0.05). There was no difference in the net scores between the 20th, 32nd, and 36th hours (*ps*>0.05).

### 3.5 Mediation analysis of repeated measurement data

**Figure 5:**
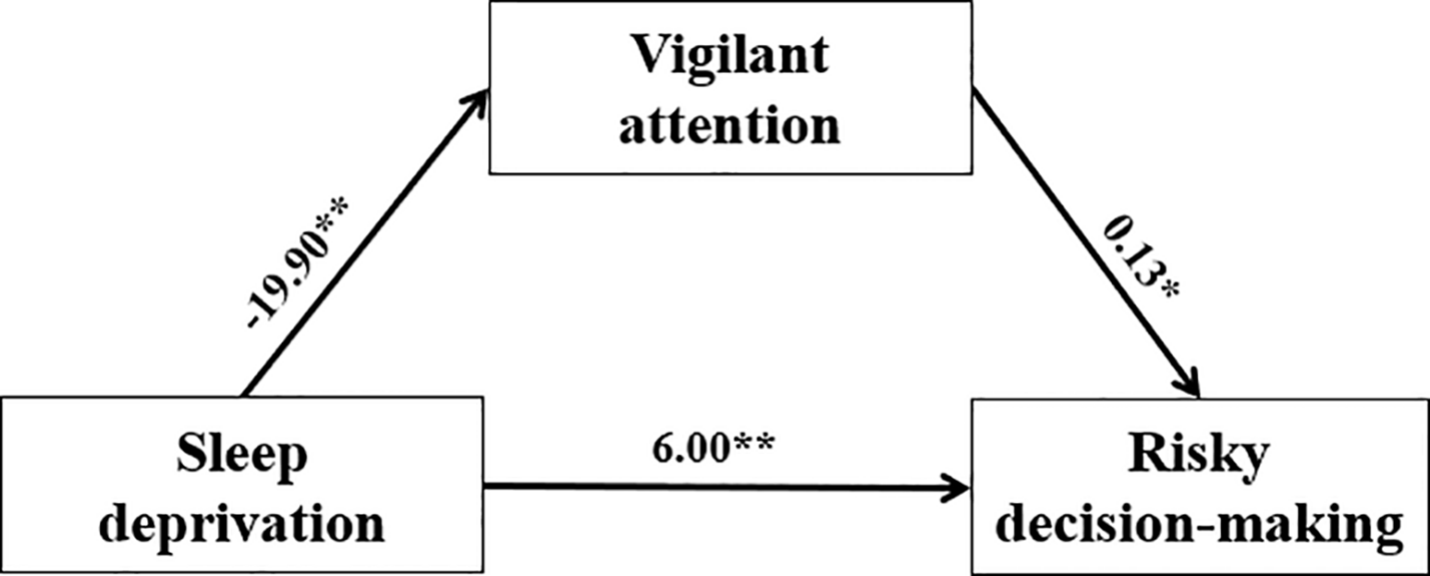
The mediating effects of vigilant attention on SD and risk decision-making using repeated measurements. SD was divided into baseline and 36th hours. Vigilant attention used with RTs of PVT indicator. Risky decision-making used with the net score of GDT indicator. Vigilant attention acted as a mediator between SD and risky decision-making. *p<.05, **p<01.

A mediation analysis further confirmed that the level of SD impacted risk decision-making through decreasing vigilant attention (Sobel test, z = -1.97, *p <*0.05, partial mediation; **Figure 5**). A bias-corrected bootstrap resampling analysis (5,000 resamples) of the effect size indicated that the mediator effect was different from zero with 95% confidence ([-6.00, -0.30]).

## 4 Discussion

The purpose of the study was to investigate the effect of SD on risky decision-making. We found that SD increased feelings of sleepiness, fatigue, and negative emotions, and decreased positive emotions. Meanwhile, SD also resulted in reduced vigilant attention, while prompting a tendency towards risky decision-making. Notably, although participants maintained their capacity to process positive feedback, their ability to utilize negative feedback declined even after just 8 hours of sleep deprivation. Additionally, vigilant attention served as a mediator in the relationship between SD and risky decision-making.

Consistent with previous studies (47, 63–65), we found that fatigue, sleepiness, pressure, and negative emotions rose significantly as the loss of sleep increased, but positive emotions decreased. SD is linked to considerably higher levels of subjective vigor, fatigue, and depression, as measured by the POMS (65). Emotional states are strongly linked to immune responses and are also linked to sleep disorders (e.g., insomnia and lethargy) as a result of SD, highlighting the critical role of sleep on mood regulation and its relationship to immune regulation (66, 67). In a continuous SD study, negative emotions varied consistently throughout the day, being higher in the morning and evening and lower during the day, but positive emotions decreased over the course of a day and over several days (68). In the present study, the effects on positive moods were greater than those on negative moods, which suggested that the influence of the circadian phase on general negative affect is particularly strong (68). There was a linear relationship between SD and mental states and moods over a 24-hour period. But after 24 hours, the individual’s mental state and mood reached a peak, remained relatively stable, and even slightly decreased. The individuals’ varied mental states and emotions showed linear and non-linear shifts while staying awake for 36 hours (44), which might be attributed to circadian factors (69).

In the study, after 36 hours of SD, PVT-RTs increased, reflecting vigilant attention decreased. SD leads to significant impairment of vigilant attention and accelerates alertness decline (70, 71). Interestingly, we found that after 24 hours, vigilant attention was slightly restored. Attention tended to decline during the first day after SD, but it was to recover the next day (68). The study began at 7 a.m., and 24 hours later, it was also morning, maybe due to biorhythms that restored attention. The temporal dynamics of vigilant attention deficits across hours and days are influenced by physiological processes, specifically sleep homeostasis and circadian regulation (69).

In the context of decision-making under risk, GDT net scores and the frequency of negative feedback decreased as SD increased, reflecting risk propensity increasing. Interestingly, we found that the influence of SD on individuals’ responsiveness to negative feedback manifested significantly after 8 hours of SD. This dynamic change declined rapidly at first and then slowly. As the duration of SD increases, their ability to effectively utilize negative feedback in evaluating risks diminishes. Previous research has revealed that risky decision processing is divided into two systems: cognitive analysis and emotional heuristics (72–74), especially in GDT (60). Attention, memory, thinking, and other cognitive capabilities must work together to make risky choices. The rational-analysis system of risky decision-making is affected by insufficient cognitive resources and reduces the performance of decision-making (75). Meanwhile, we found that SD led to decreased sensitivity to negative feedback. This suggests that participants’ sensitivity to negative feedback decreased as SD increased. Consequently, participants tended to depend more on their intuition while making decisions. When people rely too much on their intuition, they frequently have a propensity to ignore or undervalue the dangers that could come with losses because they are more concerned with the possibility of maximizing rewards (e.g., earning points in the GDT). Hence, after SD, with reduced risky awareness, participants pay more attention to potential rewards and focus more on maximizing gains, while their sensitivity to losses diminishes (22).

We constructed the mediating effect model with vigilant attention, positive emotion, and negative emotion, respectively. SD could directly predict risky decisions. We found that vigilant attention acted as a mediator between SD and risky decision-making. It seems that when people lose sleep, their vigilant attention declines, leading to a higher risk bias. Some research suggests that learning from feedback is necessary for advantageous performance in decision-making tasks (9, 76, 77). In a decision task, blunted reactions to feedback while sleep-deprived underlie failures to adapt to uncertainty and changing contingencies (78). So, due to the decrease in alertness and continuous attention, individuals may be unaware of risks and changes in the environment, which leads to risk preference. This result was only for exploratory research; the future should be on the basis of expanding the sample and controlling the related factors to further explore the result.

The number of participants collected in this study is limited due to the long experiment period and numerous test items. Therefore, due to the sample size, the effect that vigilant attention mediates the effects of sleep deprivation on risky decision-making is exploratory. The number of participants should be expanded to test the effect in future comparable investigations. The subjective scale is relatively sensitive within subjects, but the difference between subjects in the same test is not large, so the difference between before and after is small, and thus the correlation with other indicators is reduced. Thus, objective and more flexible indicators must be chosen. In addition, in this study, the subjects were college students, and the ecological validity was low. Future research should be closer to reality, recruit workers, doctors and other groups, and expand external validity. Despite these shortcomings, this study observed a dynamic effect of sleep deprivation on negative feedback in risky decision-making, which appeared after 8 hours of SD. More and more jobs, such as doctors, workers, and pilots, are required to work overtime and even reverse day and night in high-risk work (79–82). Our findings suggest that these groups may be at risk for ignoring warnings due to SD and reduce risky behavior by intervening with negative feedback cues.

## 5 Conclusions

In summary, this study has identified that sleep deprivation has a detrimental effect on individuals’ arousal, emotional state, and vigilant attention, and this effect is consistently seen 15 to 20 hours after sleep deprivation. Furthermore, sleep deprivation influences risk decision-making ability, with negative feedback processing being particularly affected even at 8 hours of deprivation. Additionally, it seems possible that vigilant attention served as a go-between for SD and risky decision-making. Taken together, these results suggest that an inability to utilize negative feedback may contribute significantly to poor decision-making in the context of sleep deprivation. Future intervention studies aimed at reducing the incidence of accidents for specific populations, such as shift workers, should focus on how to improve individuals’ ability to utilize negative feedback information when they are facing dynamic real-world situations that involve high-risk tasks.

## Data Availability

The raw data supporting the conclusions of this article will be made available by the authors, without undue reservation.
